# Healthcare Utilization, Costs of Care, and Mortality Among Patients With Spinal Muscular Atrophy

**DOI:** 10.36469/63185

**Published:** 2019-12-02

**Authors:** Hiangkiat Tan, Tao Gu, Er Chen, Rajeshwari Punekar, Perry B. Shieh

**Affiliations:** * Institution: HealthCore, Inc., Wilmington, DE; ‡ Institution: Genentech, Inc., South San Francisco, CA; †† Institution: Department of Neurology, University of California Los Angeles School of Medicine

**Keywords:** mortality, healthcare resource utilization, costs of care, treatment patterns, spinal muscular atrophy

## Abstract

**Objectives:**

To understand treatment patterns, healthcare resource utilization, and costs of care among patients with spinal muscular atrophy (SMA). Methods: SMA patients were identified from a large managed care population using administrative claims data from January 2006 to March 2016. Patients were classified into infantile, childhood-onset, and late-onset groups based on age of first SMA diagnosis. They were matched 1:1 to non-SMA patients based on age, gender, geography, and health plan type.

**Results:**

In the infantile group, 17.4% and 26.1% were treated with invasive and non-invasive ventilation, respectively. Uses of orthotics/orthoses and orthopedic surgery were frequent: 54.5% and 22.7% childhood group; 27.0% and 38.5% late-onset group. Mean per member per month costs in SMA vs. matched non-SMA patients was $25,517 vs. $406 (infantile); $6,357 vs. $188 (childhood-onset); $2,499 vs. $742 (late-onset).

**Conclusions:**

SMA patients, particularly with infantile onset, incurred significantly higher healthcare utilization and costs than the general population.

## INTRODUCTION

Spinal muscular atrophy (SMA) is rare, occurring in approximately 1 in 10 000 newborns, 1–3 but it is associated with considerable clinical, psychosocial, and economic burden.^4^–^9^ SMA is an autosomal recessive disease characterized by progressive degeneration of the motor neurons, resulting in atrophy of skeletal muscles and generalized weakness.^1^–^3^ The most common form of SMA is caused by mutations in the *SMN1* gene on chromosome 5q, which result in a relative deficiency of SMN protein. It is typically classified according to age of onset and degree of disability.^10^–^13^ Type I is typically diagnosed before 6 months of age, and infants are unable to sit unsupported; Type II is typically diagnosed between 6 months and 2 years of age in children who are able to sit independently but never develop to ability to ambulate.^9^–^11^ Type III is typically diagnosed between 18 months and 3 years of age, but may also be diagnosed into the teens. This is a less severe type of SMA with patients able to ambulate, but they become progressively weaker and their mobility becomes increasingly limited.^9^–^11^ Some clinicians also recognize Type IV SMA, where symptoms develop in adults and diagnosis is often made later in life. Type IV is uncommon but is often diagnosed clinically; these patients may have motor neuron diseases not associated with *SMN*1 mutations.^9^–^11^ The disease burden varies by type.^5^,^12^ For example, if left untreated, children with Type I SMA would have respiratory insufficiency and require pulmonary support and inpatient care before dying within the first 2 years.^4^,^12^–^15^ Those with Type II SMA have less need for inpatient care but greater use of medications than those with infantile SMA; patients with Type III or IV SMA are primarily burdened with functional deficiencies and benefit from physical therapy, orthopedic surgery, or other medical devices.^4^,^12^–^14^

Given the rarity of SMA, published research is based on small clinical samples. The economic research that has been published describes healthcare costs substantially higher for patients with SMA compared with a healthy population, particularly for infantile SMA.^4^ Including both direct and indirect costs, per capita expenditures for infantile SMA exceed $184 000, which is similar to other chronic disabling conditions.^14^–^16^ Mean direct costs were found to be 14 times higher for patients with SMA compared with average healthcare expenditures in Germany,^17^ and direct and indirect SMA-related costs top $950 million in the US.^18^ These economic studies, however, were limited: some sample sizes were small – even as few as 7 patients^18^ – and several studies were conducted in Europe, with a caveat that the results were likely not generalizable to other countries.^17^ Armstrong and colleagues studied SMA-related costs and resource utilization using data from the US Department of Defense Military Healthcare System, which only included active duty and retired military personnel and their dependents.^4^ The Lewin Group researched SMA costs using administrative data but did not include a control group without SMA.^15^ Without a healthy comparison group, it is difficult to accurately assess the incremental burden of SMA. A recently published retrospective analysis using the Kids’ Inpatient Database concluded the average daily hospitalization charge for children with infantile SMA was almost twice that for children with no chronic conditions.^8^ Considering children with infantile SMA require an average of 4.2 hospitalizations per year, and each hospitalization is longer and uses more resources than those for children without a chronic condition, costs associated with SMA are actually much higher than reported by smaller studies.^8^

In December 2016, the Food and Drug Administration (FDA) approved the first disease-modifying therapy (nusinersen, Spinraza^®^) and in 2019 approved the first gene replacement therapy (onasemnogene abeparvovec-xioi, Zolgensma^®^).^19^,^20^ The emergence of new treatments is changing the management and cost burden of SMA. Assessment of the value of these new, potentially high-cost treatments requires a solid baseline understanding of the disease-related complications, supportive care, and associated medical costs.

The current study was designed to describe supportive care and healthcare resource utilization associated with SMA. The data were derived from one of the largest administrative claims databases in the US, providing the means to describe the natural history and outcomes of patients with SMA in greater numbers than have been previously described. Patients with SMA were matched to a general population without SMA to allow for meaningful comparisons and quantifying the incremental burden of SMA. This study was conducted before the approval of the first treatment for SMA, thereby providing important baseline data on care patterns and costs.

## METHODS

This retrospective cohort study was conducted using the HealthCore Integrated Research Database^®^ (HIRD^®^) to identify patients with SMA between January 1, 2006 and March 31, 2016. The HIRD is a repository of clinically rich and adjudicated longitudinal medical, pharmacy, and eligibility data originating in 14 geographically dispersed US health plans. Administrative claims provide a record of all healthcare expenditures reimbursed through insurance, including hospitalization, outpatient office visits, and pharmacy costs. Expenses paid directly by the patient, such as copays or over-the-counter medications, are not captured in claims. The HIRD includes a nationally representative database in terms of age and gender, although adults older than 65 years are under-represented. At the time of the study, the database had access to administrative claims for 37.7 million members.^21^ The US Social Security Death Index (SSDI), which is a database of all deaths reported to the US Social Security Administration, was used to capture mortality. Unique patient identifiers were used to match records in the HIRD to those in the SSDI database to identify deceased patients and dates of death. This was operated fully in compliance with the Health Insurance Portability and Accountability Act. Only de-identified patient data were accessed and the study was exempt from Institutional Review Board review.

### INCLUSION CRITERIA

Patients were included in the study if they had two or more medical claims on two distinct service dates, at least 30 days apart, with SMA-related diagnoses (ICD-9 diagnosis codes: 335.0x, 335.1x, ICD-10 diagnosis codes: G12.0, G12.1, GL12.8, G12.9) during the patient identification period from January 1, 2006 to March 31, 2016.

The date of the first SMA-related diagnosis was set as the index date for patients with SMA. In the clinical setting, infants with SMA might have been diagnosed with hypotonia prior to being diagnosed with SMA. Therefore, for patients with one or more hypotonia-related diagnosis (ICD-9 diagnosis code: 358.8x, 359.0x, 359.21–359.23, 359.29; ICD-10 diagnosis codes: G71.11 – G71.13, G71.19) before the age of 6 months, the index date was set as the service date of the first SMA-related or hypotonia-related diagnosis. The baseline period was defined as the 12 months prior to the index date. For infants younger than 1 year, the baseline period was defined as the period between the enrollment start date and the index date.

This study used the age of first occurrence of SMA-related diagnosis to categorize patients into infantile, childhood onset, or late onset cohorts. The infantile cohort included infants with their index date before the age of 6 months, while children in the childhood onset cohort had their index date between the ages of 6 months and 3 years. Patients who did not fall into the prior two cohorts were regarded as the late onset cohort.

All patients except infants were required to have continuous health plan enrollment for a minimum of 12 months prior to the index date. This requirement maximized the chances that the first observed SMA-related diagnosis was in fact the first time they were diagnosed. For infants with index date less than 1 year, continuous health plan enrollment beginning 30 days after birth was required. Patients were followed from the index date to the end of the study period, the end of health plan eligibility, or death, whichever occurred first. Patients with a diagnosis of amyotrophic lateral sclerosis (ALS) were excluded from the late onset cohort to minimize potential confounding by ALS, as both diseases share common symptoms and are difficult to differentiate with confidence using administrative data alone without medical records.

Patients with no SMA diagnoses, but with the rest of inclusion/exclusion criteria, were exactly matched with 1:1 ratio to patients with SMA based on birth year and month, gender, health plan type, region, and health plan subscriber status. The index date for patients without SMA was assigned based on similar distribution of lag time between the start of enrollment and the index date of the matched patient with SMA.

### STUDY OUTCOMES

The primary outcome was utilization of supportive care–including pulmonary, gastrointestinal and nutrition, and orthopedic care–measured through medication use, healthcare resource utilization, costs of care, and mortality rates. Healthcare resource utilization included inpatient, emergency department, physician office, occupational/physical therapy, or home health visits, and pharmacy prescriptions. Cost of care was the sum of health plan-paid costs, patient-paid costs, and coordination of benefits, if any. Costs were adjusted to 2015 dollars based on consumer price index (CPI) medical care index information provided by the Bureau of Labor Statistics. All the costs were reported as per-member-per-month (PMPM). Only direct medical and pharmacy costs were collected; indirect expenses such as transportation to appointments or respite care were not captured in claims. Mortality was derived using the SSDI. The number of deaths and time to death were presented for each cohort.

### STATISTICAL ANALYSIS

Descriptive statistics included means, medians, standard deviation (SD), and interquartile range (IQR) for continuous measures and relative frequencies for categorical measures. The statistical significance between SMA and matched non-SMA cohorts was assessed using the Wilcoxon signed-rank test for continuous variables, while the McNemar test or Pearson χ^2^ test was used for categorical variables. Generalized linear model (GLM), with log link and gamma distribution, was used to estimate the incremental costs of SMA while adjusting for the length of follow-up (and Deyo-Charlson comorbidity index for late onset cohort). Kaplan-Meier analysis was conducted for mortality risk comparison.

## RESULTS

**Figure 1:**
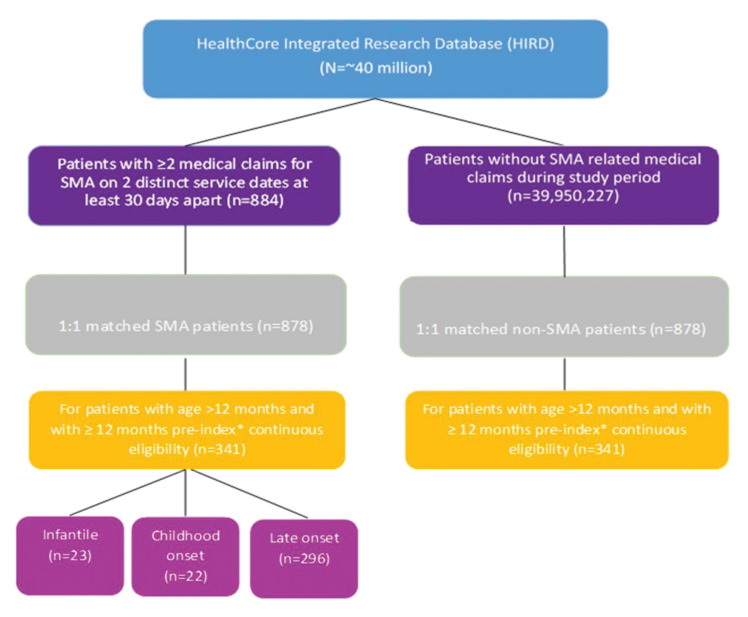
Patient Flow Chart

A total of 341 pairs of patients with and without SMA were included in the study, 23 in the infantile cohort, 22 in the childhood onset cohort, and 296 in the late onset cohort (Figure 1). The mean age was 0.2 years (±0.14 years) in the infantile cohort; 0.9 years (±0.87 years) in the childhood onset cohort; and 52.2 years (±21.95 years) in the late onset cohort (Table 1). All cohorts had slightly more males than females (52.2% infantile cohort; 63.6% childhood onset cohort; 54.4% late onset cohort). The mean length of follow up ranged from 11.0 months in the infantile SMA cohort to 36.0 months in the late onset cohort.

### MEDICATION UTILIZATION

During follow up, acid suppressants and antibiotics were the most frequently prescribed medications of interest for patients in the infantile (56.5% each) cohort, whereas antibiotics were most common in the childhood onset (68.2%) and late onset (67.2%) cohorts (Table 2). Other frequently prescribed medications in the late onset cohort included muscle relaxants (36.8%), benzodiazepines (36.1%), and antidepressants (31.8%). In the childhood onset cohort, muscle relaxants and benzodiazepines (31.8% each) were also frequently prescribed.

### HEALTHCARE RESOURCE UTILIZATION

Pulmonary services were used by a large proportion of patients in the infantile cohort, particularly pulmonologist office visits, swallow function evaluation, and mechanically assisted cough devices (Table 3). Invasive ventilation was used by 17.4% of those in the infantile cohort; 26.1% had noninvasive ventilation. All cohorts used orthopedic care, namely radiologic testing (95.7% infantile cohort; 81.8% childhood onset cohort; 83.4% late onset cohort) and evaluation by a physical or occupational therapist (65.2% infantile cohort; 68.2% childhood onset cohort; 50.3% late onset cohort). A majority of patients in the childhood onset cohort used physical or occupational therapy (86.4%) and orthotics or orthoses (54.5%). Although a majority of patients in the late onset cohort used physical or occupational therapy (62.2%), orthotics or orthoses were less common (27.0%). Neurologists made the majority of specialist visits in the infantile (69.6%) and childhood onset cohorts (81.8%). In the late onset cohort, physician visits were roughly divided between neurologists (41.6%) and primary care physicians (53.0%).

Hospitalizations and office visits during the follow-up period were higher among patients with SMA than in the matched patients without SMA. The proportion of inpatient hospitalizations between patients with SMA and matched patients without SMA were 91.3% vs 17.4%, respectively, in the infantile cohort; 50.0% vs 13.6%, respectively, in the childhood onset cohort; and 37.2% vs 18.2%, respectively, in the late onset cohort (Table 4). The annualized mean number of physician office visits was also higher for each of the

SMA cohorts compared with the non-SMA matched cohorts: 24.0 (±15.16) vs 6.8 (±5.13), respectively, in the infantile cohort; 15.2 (±8.70) vs 5.6 (±4.26), respectively, in the childhood onset cohort; and 11.8 (±14.70) vs 5.8 (±6.03), respectively, in the late onset cohort. Similar results were observed for the proportion of occupational or physical therapy visits between the SMA and non-SMA cohorts: 47.8% vs 4.3%, respectively, in the infantile cohorts; 86.4% vs 4.5%, respectively, in the childhood onset cohort; and 54.0% vs 25.5%, respectively, in the late onset cohort.

### COSTS OF CARE

**Figure 2:**
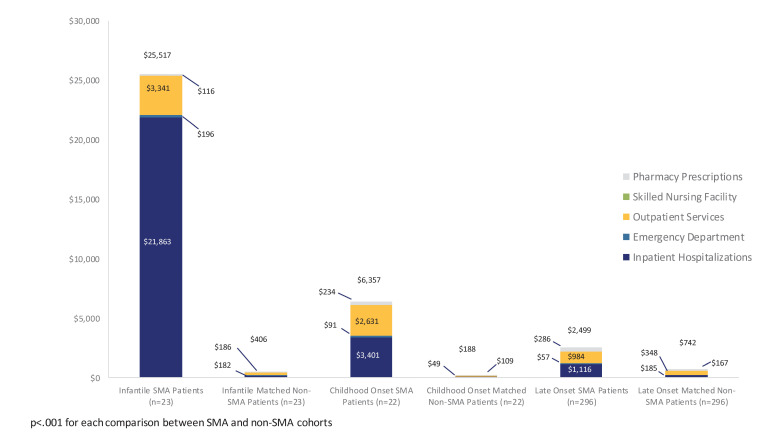
Per-Member-Per-Month Mean All-Cause Costs Between SMA and Matched Non-SMA Patients

Total mean healthcare costs, on a per-patient-per-month (PMPM) basis, appeared highest in the infantile SMA cohort (mean $25 517; SD $29 777; median $13 347), followed by the childhood (mean $6357; SD $8938; median $2259) and late onset (mean $2499; SD $5018; median $953) SMA cohorts. In the non-SMA cohorts, total mean healthcare costs appeared highest in the late onset cohort (mean $742; SD $1566; median $238) followed by the infantile (mean $406; SD $558; median $162) and childhood (mean $188; SD $325; median $108) cohorts (Figure 2). After further adjusting for length of follow up, patients in the infantile SMA cohort had on average 54.2 times higher (95% CI 27.2 – 107.9, p<0.001) total healthcare costs than matched patients without SMA. The SMA childhood onset cohort had on average 33.7 times higher total healthcare costs (95% CI: 15.9 – 71.6, p<0.001) than the matched non-SMA patients. The mean total healthcare costs in the late onset SMA cohort were 3.1 times higher (95% CI 2.5 – 3.8, p<0.001) than those of the matched non-SMA cohort.

### MORTALITY

The mortality rate in the infantile SMA cohort was 17.3%, followed by 4.5% in the childhood and 5.4% in the late onset SMA cohorts (Table 5). In contrast, no deaths occurred in the non-SMA matched infantile and childhood cohorts, and the mortality rate in the late onset non-SMA matched cohort was 2.3%. The mean number of months from index date to death was shortest in the infantile SMA cohort (6.7 months [±6.11]). The mean number of months from index date to death was 19.1 in the childhood cohort and 26.2 (±19.75) in the late onset cohort. The mean number of months from index date to death in the non-SMA matched late onset cohort was 36.4 months (±23.13).

**Figure 3:**
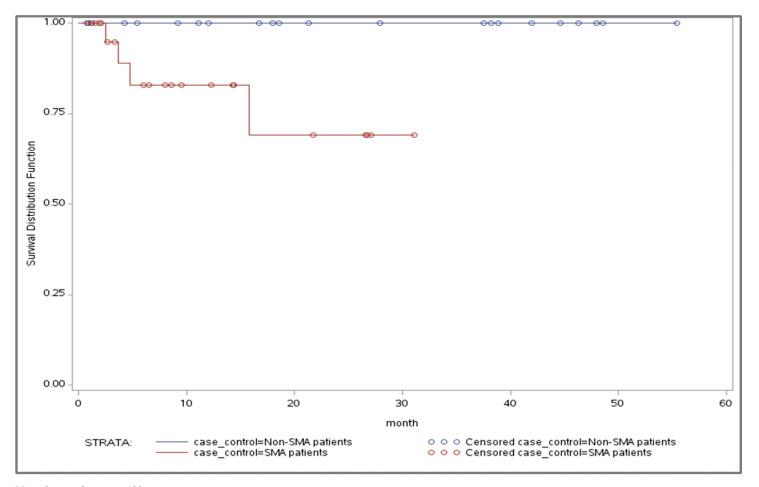
Kaplan-Meier Curve of Mortality: Infantile SMA and Matched Non-SMA Note: log-rank test p=0.0193

Kaplan-Meier analysis showed a significant difference in mortality risk between the infantile SMA and matched non-SMA cohorts (log rank p=0.0193; Figure 3). No significant differences in mortality risk were found between the SMA and non-SMA childhood onset and late onset cohorts, respectively.

## DISCUSSION

This retrospective observational study found significant healthcare resource utilization and cost burden of SMA, particularly among those with infantile onset SMA. The study also provided an important baseline on how patients with SMA were being managed with various supportive care services and medications in the healthcare system prior to the first approved disease-modifying treatment.

Use of supportive care services, including orthopedic and pulmonary services, varied among the three cohorts, as did the types of physicians patients visited. Between-group differences in utilization of supportive care services reflect the varying needs of the cohorts.^22^ Whereas the youngest children with the most severe form of SMA require respiratory support and other intensive care, older patients with less severe disease need functional support, such as braces, orthotics, and physical therapy.^4^,^15^,^22^–^24^

Total healthcare costs also reflected the varying needs of the cohorts: inpatient hospitalizations accounted for the bulk of the costs in the infantile cohort, while prescription medication costs contributed most to total costs in the childhood onset cohort. These findings were consistent with previous analyses of the economic burden of SMA, which not only reported higher costs associated with infantile SMA, but also attributed a greater proportion of total costs in the infantile SMA group to inpatient expenses.^4^,^8^,^9^

As expected, the mortality rate was highest in the infantile cohort; however, the mean survival time of 6.7 months was slightly lower than the 8 months reported in previous research.^25^ Furthermore, the 17.3% mortality rate in the infantile cohort over an average follow-up of 11 months appears low, but may be due to patients receiving mostly aggressive supportive care.

In addition, SMA has a significant disease burden not only on the lives of patients, but also on their families, caregivers, and the broader society. In a survey commissioned by the Muscular Dystrophy Association (MDA), 16 to 24 hours of daily care is needed for more than 80% of patients diagnosed before 3 years of age, and 50% for other patients with SMA.^14^ Families with patients diagnosed before 3 years of age were reported to have an annual cost of $50 542 associated with moving or modifying homes, purchasing motor vehicles, professional caregiving, and other non-medical costs.^14^ SMA is also associated with substantial indirect costs. In a survey of adult patients with Type II SMA, less than half were employed.^26^ Many of the parents of patients with SMA must stop working at some point in their lives to take care of their loved ones.^14^ Although non-medical and indirect costs might constitute a significant portion of the societal cost of SMA, these costs are not captured in claims and were not included in this study.

This study had several limitations. Given the nature of rare disease of SMA, the sample sizes for the infantile and childhood cohorts were small and the results should be interpreted with caution. Subcategories of SMA could not be distinguished with specific markers or ICD-9 codes. It was possible that some patients with childhood onset SMA were misclassified to the late onset cohort. All patients in the study were members of a large commercial health plan. The results might not be generalizable to patients with other types of health insurance, such as government-sponsored health plans, uninsured, or underinsured. A number of patient demographics (e.g., ethnicity), confirmation of SMA subtype, disease prognosis, and ambulation status were not available in the administrative data. Additionally, indirect patient expenses as well as out-of-pocket expenses are not captured in administrative claims and thus the costs may have been underestimated. Lastly, the administrative data might be subject to measurement error due to miscoding. Such error was likely to be random between groups, and unlikely to influence the incremental estimates while comparing SMA and non-SMA groups.

The study time horizon predated the introduction of the first treatment for SMA, therefore, none of the patients in this study received any treatment. An updated burden of illness analysis, reflecting the use of new treatment in patients with SMA and specifically patients with different SMA subtypes, will be needed to understand the impact of new treatment on patient outcomes, care delivery, and costs in the healthcare systems.^9^ This study could be used as a reference point for future research.

### CONCLUSIONS

This study found patients with SMA, particularly with infantile onset, incurred substantial healthcare utilization and costs of care. The study also provided an important baseline on how patients with SMA were being managed in the healthcare system with various supportive care services and medications prior to the first approved treatment.

## Figures and Tables

**Table 1:** Baseline Demographic Characteristics Among Matched SMA and Non-SMA Patients

	Infantile^a^ SMA Patients (n=23)	Infantile Matched Non-SMA Patients (n= 23)	Childhood Onset^b^ SMA Patients (n=22)	Childhood Onset-Matched Non-SMA Patients (n= 22)	Late Onset^c^ SMA Patients (n=296)	Late Onset-Matched Non-SMA Patients (n= 296)
Age (yrs), mean (SD)	0.2 (±0.14)	0.2 (±0.14)	0.9 (±0.87)	0.9 (±0.87)	52.2 (±21.95)	52.1 (±21.90)
Male, %	52.2	52.2	63.6	63.6	54.4	54.4
Post-index enrollment (months), mean (SD)	11.0 (±9.58)	23.8 (±18.5)	27.7 (±23.37)	33.5 (±32.04)	36.0 (±26.50)	36.3 (±29.81)

SD=standard deviation; yrs=years

a Infantile: patients with their first SMA-related ICD 9 diagnostic codes: 335.0x, 335.1x; ICD-10 diagnosis codes: G12.0, G12.1, G12.8, G12.9) medical claim before the age of 6 months; OR patients with ≥1 hypotonia-related (ICD-9 diagnostic codes: 358.8x, 359.0x, 359.21–359.23, 359.29; ICD-10 diagnosis codes: G71.11–G71.13, G71.19) medical claims (before the age of 6 months) followed by ≥1 SMA-related medical claims

b Childhood onset: patients who did not meet the infantile group criteria AND with their first SMA-related medical claim between 6 months and 3 yrs

c Late onset: patients with SMA who were not included in infantile or childhood onset categories

**Table 2:** Concomitant Medication Use During Follow Up Among Patients With SMA

	Infantile^a^ (n=23)	Childhood Onset^b^ (n=22)	Late Onset^c^ (n=296)
Length of post-index follow-up, months, mean (SD)	11.0 (±9.58)	27.7 (±23.37)	36.0 (±26.5)
Acid suppressants	56.5%	31.8%	28.7%
Antibiotics	56.5%	68.2%	67.2%
Muscle relaxants	17.4%	31.8%	36.8%
Benzodiazepines	17.4%	31.8%	36.1%
Prokinetic agents	13.0%	4.5%	3.4%
Anti-anxiety	8.7%	9.1%	21.3%
Antidepressants	0.0%	0.0%	31.8%
Botulinum toxin	0.0%	0.0%	2.4%
CNS stimulants	0.0%	0.0%	6.4%

CNS=central nervous system; SD=standard deviation

a Infantile: patients with their first SMA-related (ICD-9 diagnostic codes: 335.0x, 335.1x; ICD-10 diagnosis codes: G12.0, G12.1, G12.8, G12.9) medical claim before the age of 6 months; OR patients with ≥1 hypotonia-related (ICD-9 diagnostic codes: 358.8x, 359.0x, 359.21–359.23, 359.29; ICD-10 diagnosis codes: G71.11 G71.13, G71.19) medical claims (before 6 months of age) followed by ≥1 SMA-related medical claims

b Childhood onset: patients who did not meet the infantile group criteria AND with their first SMA-related medical claim between 6 months and 3 yrs of age

c Late onset: patients with SMA who were not included in infantile or childhood onset categories

**Table 3:** Supportive Care Utilization During Follow Up Among Patients With SMA

	Infantile^a^ (n=23)	Childhood Onset^b^ (n=22)	Late Onset^c^ (n=296)
Length of post-index follow-up, months, mean (SD)	11.0 (±9.58)	27.7 (±23.37)	36.0 (±26.5)
Pulmonary, n (%)			
Swallow function evaluation^d^	56.5%	31.8%	9.8%
Mechanically assisted cough devices^e^	52.2%	31.8%	1.4%
Pulmonologist office visits^d^	52.2%	36.4%	13.5%
Polysomnography/sleep studies^d^	21.7%	36.4%	12.2%
Noninvasive ventilation^e^	26.1%	9.1%	4.1%
Invasive ventilation^e^	17.4%	13.6%	1.0%
Pulmonary function testing^d^	13.0%	45.5%	14.5%
Chest physiotherapy	0.0%	0.0%	0.3%
Gastrointestinal/nutritional care, n (%)			
Enteral feeding products^e^	60.9%	39.4%	5.1%
Videofluoroscopic swallow studies (modified barium swallow studies)^d^	43.5%	13.6%	9.8%
Gastrostomy tube placement^e^	34.8%	13.6%	2.0%
Laparoscopic anti-reflux procedures (eg, Nissen fundoplication)^e^	21.7%	13.6%	0.0%
Nasogastric/nasojejunal tube placement^e^	4.3%	4.5%	0.7%
Speech/occupational therapy^e^	4.3%	0.0%	4.1%
GI motility studies^d^	0.0%	0.0%	0.7%
Speech/occupational therapist evaluation^d^	0.0%	0.0%	0.7%
Orthopedic care, n (%)			
Radiologic testing^d^	95.7%	81.8%	83.4%
Physical/occupational therapist evaluation^d^	65.2%	68.2%	50.3%
Physical/occupational therapy^e^	47.8%	86.4%	62.2%
Orthotics/orthoses^e^	30.4%	54.5%	27.0%
Orthopedic surgery^e^	17.4%	22.7%	38.5%
Durable medical equipment (eg, wheelchairs, walkers)^e^	13.0%	45.5%	14.5%
Orthopedic physician evaluation^d^	13.0%	36.4%	35.1%
Scoliosis correction surgery^e^	4.3%	4.5%	2.0%
Visits, n (%)			
Neurologist	69.6%	81.8%	41.6%
Home care services	13.0%	4.5%	4.7%
Primary care physician	8.7%	27.3%	53.0%

CNS=central nervous system; SD=standard deviation

a Infantile: patients with their first SMA-related (ICD-9 diagnostic codes: 335.0x, 335.1x; ICD-10 diagnosis codes: G12.0, G12.1, G12.8, G12.9) medical claim before the age of 6 months; OR patients with ≥1 hypotonia-related (ICD-9 diagnostic codes: 358.8x, 359.0x, 359.21–359.23, 359.29; ICD-10 diagnosis codes: G71.11–G71.13, G71.19) medical claims (before 6 months of age) followed by ≥1 SMA-related medical claims.

b Childhood onset: patients who did not meet the infantile group criteria AND with their first SMA-related medical claim between 6 months and 3 yrs of age

c Late onset: patients with SMA who were not included in infantile or childhood onset categories

d Patients with evaluation

e Patients with treatment

**Table 4:** Follow Up All-Cause Healthcare Utilization^a^ Among Patients With SMA

	Infantile^b^ SMA Patients (n=23)	Infantile Matched Non-SMA Patients (n= 23)	Childhood Onset^c^ SMA Patients (n=22)	Childhood Onset-Matched Non-SMA Patients (n= 22)	Late Onset^d^ SMA Patients (n=296)	Late Onset-Matched Non-SMA Patients (n= 296)
Length of post-index follow-up (months), mean (SD), median	11.0 (± 9.58), 8.0	23.8 (± 18.50), 18.6	27.7 (± 23.37), 24.0	33.5 (± 32.04), 21.1	36.0 (± 26.50), 29.6	36.3 (± 29.81), 28.6
Inpatient hospitalizations						
Patients with ≥1, %	91.3%	17.4%	50.0%	13.6%	37.2%	18.2%
Length of stay among patients with ≥1 inpatient hospitalization (days), mean (SD), median	40.8 (± 40.39), 19.6	13.7 (± 12.96), 12.8	14.9 (± 15.92), 6.0	0.7 (± 0.89), 0.3	9.1 (± 18.03), 2.1	3.4 (± 4.07), 1.5
Emergency department visits						
Patients with ≥1, %	69.6%	21.7%	40.9%	22.7%	37.2%	25.8%
Outpatient office visits						
Number per patient among all patients, mean (SD), median	24.0 (± 15.16), 18.5	6.8 (± 5.13), 5.6	15.2 (± 8.70), 12.9	5.6 (± 4.26), 5.9	11.8 (± 14.70), 8.5	5.8 (± 6.03), 3.9
Occupational/physical therapy-related visits						
Patients with ≥1 visits, %	47.8%	4.3%	86.4%	4.5%	54.0%	25.5%
Home health visits						
Patients with ≥1 visits, %	13.0%	0.0%	4.5%	0.0%	4.1%	1.2%
Durable medical equipment (wheelchair, walkers, etc)						
Patients with ≥1 piece of equipment, %	13.0 %	0.0%	45.5%	0.0%	12.6%	0.6%
Pharmacy prescriptions						
Number of prescriptions per patient among all patients, mean (SD), median	8.0 (± 8.20), 7.5	2.7 (± 4.59), 1.1	12.0 (± 12.27), 7.3	2.3 (± 3.20), 0.9	19.4 (± 18.11), 14.4	12.1 (± 15.82), 6.1

a Only utilization counts are annualized; number and percentage of patients are not annualized.

b Infantile: patients with their first SMA-related (ICD-9 diagnostic codes: 335.0x, 335.1x; ICD-10 diagnosis codes: G12.0, G12.1, G12.8, G12.9) medical claim before the age of 6 months; OR patients with ≥1 hypotonia-related (ICD-9 diagnostic codes: 358.8x, 359.0x, 359.21–359.23, 359.29; ICD-10 diagnosis codes: G71.11–G71.13, G71.19) medical claims (before the age of 6 months) followed by ≥1 SMA-related medical claims

c Childhood onset: patients who did not meet the infantile group criteria AND with their first SMA-related medical claim between 6 months and 3 yrs

d Late onset: patients with SMA who were not included in infantile or childhood onset categories

**Table 5:** Mortality During Follow Up Among Patients With SMA

	Infantile^a^ SMA Patients (n=23)	Infantile Matched Non-SMA Patients (n=23)	Childhood Onset^b^ SMA Patients (n=22)	Childhood Onset-Matched Non-SMA Patients (n= 22)	Late Onset^c^ SMA Patients (n=296)	Late Onset-Matched Non-SMA Patients (n= 296)
Length of post-index follow-up, months mean (SD), median	11.0 (± 9.58), 8.0	23.8 (± 18.50), 18.6	27.7 (± 23.37), 24.0	33.5 (± 32.04), 21.1	36.0 (± 26.50), 29.6	36.3 (± 29.81), 28.6
Mean time from index date to death, months (SD), median	6.7 (± 6.11), 4.3	N/A	19.1 (N/A), 19.1	N/A	26.2 (± 19.75), 20.3	36.4 (± 23.13), 32.6
Total mortality, %						
0 – 6 months post-index	13.0%	0.0%	0.0%	0.0%	1.4%	0.0%
7 – 12 months post-index	0.0%	0.0%	0.0%	0.0%	0.0%	0.0%
13 – 18 months post-index	4.3%	0.0%	0.0%	0.0%	1.0%	0.7%
19 – 24 months post-index	0.0%	0.0%	0.0%	0.0%	0.7%	0.3%
25 – 36 months post-index	0.0%	0.0%	4.5%	0.0%	0.3%	0.3%
36 months after post-index period	0.0%	0.0%	0.0%	0.0%	2.0%	1.0%

a Infantile: patients with their first SMA-related (ICD-9 diagnostic codes: 335.0x, 335.1x; ICD-10 diagnosis codes: G12.0, G12.1, G12.8, G12.9) medical claim before the age of 6 months; OR patients with ≥1 hypotonia-related (ICD-9 diagnostic codes: 358.8x, 359.0x, 359.21–359.23, 359.29; ICD-10 diagnosis codes: G71.11–G71.13, G71.19) medical claims (before the age of 6 months) followed by ≥1 SMA-related medical claims

b Childhood onset: patients who did not meet the infantile group criteria AND with their first SMA-related medical claim between 6 months and 3 yrs of age

c Late onset: patients with SMA who were not included in infantile or childhood onset categories
